# Population structure of the fish pathogen *Flavobacterium psychrophilum* at whole-country and model river levels in Japan

**DOI:** 10.1186/1297-9716-44-34

**Published:** 2013-05-17

**Authors:** Erina Fujiwara-Nagata, Céline Chantry-Darmon, Jean-François Bernardet, Mitsuru Eguchi, Eric Duchaud, Pierre Nicolas

**Affiliations:** 1 Department of Fisheries, Kinki University, Nara, Japan; 2 INRA, Virologie et Immunologie Moléculaires UR892, Jouy-en-Josas, France; 3 INRA, Mathématique Informatique et Génome UR1077, Jouy-en-Josas, France

## Abstract

The bacterium *Flavobacterium psychrophilum* is a serious problem for salmonid farming worldwide. This study investigates by multilocus sequence typing (MLST) the population structure of this pathogen in Japan where it is also a major concern for ayu, a popular game fish related to salmoniforms. A total of 34 isolates collected across the country and 80 isolates sampled in a single model river by electrofishing were genotyped. The data accounting for 15 fish species allowed identifying 35 distinct sequence types (ST) in Japan. These ST are distinct from those reported elsewhere, except for some ST found in rainbow trout and coho salmon, two fish that have been the subject of intensive international trade. The pattern of polymorphism is, however, strikingly similar across geographical scales (model river, Japan, world) in terms of the fraction of molecular variance linked to the fish host (~50%) and of pairwise nucleotide diversity between ST (~5 Kbp^-1^). These observations go against the hypothesis of a recent introduction of *F. psychrophilum* in Japan. Two findings were made that are important for disease control: 1) at least two independent *F. psychrophilum* lineages infect ayu and 2) co-infections of the same individual fish by different strains occur.

## Introduction

Diseases are an important problem in fish farming and control of bacterial pathogens relies mostly on repeated antibiotic treatments incompatible with the development of sustainable aquaculture [1]. The fish-pathogenic bacterium *Flavobacterium psychrophilum* is the causative agent of the “bacterial cold water disease” (BCWD) and the “rainbow trout fry syndrome”, two salmonid diseases that cause considerable losses in rainbow trout (*Oncorhynchus mykiss*) and coho salmon (*Oncorhynchus kisutch*) farming industries worldwide [2-5]. In Japan, *Flavobacterium psychrophilum* was first isolated in 1987 from cultured ayu (*Plecoglossus altivelis altivelis*), a fish related to salmoniforms [6]. The status of *F. psychrophilum* in the country before the development of the fish farming industry and possible importation from foreign sources is unclear [7-9] but it is currently recognized as a serious problem, in particular for ayu [10]. Indeed, to increase the stocks of this very popular game fish, juveniles hatched from artificially fertilized eggs or captured in coastal areas are released into rivers by fish farming companies and experimental stations located in each prefecture. The bacterium is then probably disseminated with ayu juveniles. Infections are now repeatedly reported not only in fish farms but also in natural environments [6,11].

Many species beyond the ayu and farmed rainbow trout and coho salmon have been reported to harbor *F. psychrophilum* in Japan. These include migrating salmonids whose geographical ranges extend throughout the north-Pacific and more local species and subspecies. The actual host range of *F. psychrophilum* is, however, not limited to salmonids [12], although the infection is apparently less severe in non-salmonids [13]. In Japan, *F. psychrophilum* has been reported on a number of local non-salmonid wild fish species [14-16].

Understanding the degree and determinants of host specificity of *F. psychrophilum* strains as well as their routes of dissemination would help setting rational practices to limit the spreading of the disease. Correlations between bacterial genotype and host fish have been observed in several studies on field isolates [14,17-20] and we recently reported that ayu could be experimentally infected in bath infection challenges by isolates collected from ayu but not from coho salmon or rainbow trout [14]. However mimicking the conditions of natural infections is generally difficult for *F. psychrophilum*[21-24] and field studies that would provide a deeper knowledge of the distribution of the genotypes in terms of geographical area and host fish species are essential.

Following the publication of the complete genome sequence of a *F. psychrophilum* strain [25], a first Multi-locus sequence typing (MLST) analysis was conducted on a set of 50 isolates collected from 11 different fish species in 12 different countries over the world [17]. This initial study identified 33 different sequence types (ST), some of which could be grouped into clonal complexes (CC) associated with particular fish species, especially rainbow trout and coho salmon. The results also revealed remarkably high rates of homologous recombination and limited nucleotide diversity. However, the small number of isolates from each particular geographical origin did not allow the description of population structure at a regional scale. A more recent analysis of 66 additional isolates from farmed rainbow trout in France [26] concluded to the predominance of a single clonal complex. In the present study we investigated the genetic diversity of *F. psychrophilum* in Japan using a higher number of isolates than for any other region and taking into account the diversity of fish hosts. We also compare the patterns of polymorphism at the world, country and single model river levels.

## Materials and methods

### Field sampling of *F. psychrophilum* isolates

A first set of Japanese *F. psychrophilum* isolates was contributed by correspondents all over Japan (listed in Additional file 1). A second set of isolates was collected in the downstream area of the ~9.5 km long Chinai River near (~0.3 km) its mouth into Lake Biwa, the largest lake in Japan. With an authorization of the Shiga prefecture, fish were caught by electrofishing, kept at low temperature into tanks, quickly transported to our laboratory and euthanized by severing their spinal cord. Gill samples were cut into pieces of ~25 mm^2^ and suspended in 1 mL of phosphate buffered saline (PBS). The tubes were vortexed during 30 s at maximum speed and the suspensions were further diluted in PBS as to obtain well-separated colonies on modified cytophaga (MCYT) agar [27]. Samples from internal organs and skin lesions were collected using a sterile loop and streaked directly onto MCYT plates. Water samples (containing 2000 ~ 3000 cfu mL^-1^) were diluted to 10^-4^ in PBS prior to being spread onto MCYT plates. Caddisfly cases were vigorously vortexed in 1.5 mL of PBS and the suspensions were diluted and spread onto MCYT plates. All plates were incubated at 15°C for 4 days. Yellow colonies were selected for identification as *F. psychrophilum* based on the PCR amplification of the 16S rRNA and *gyrB* genes [18,28,29] according to the guidelines of the council for the control of bacterial cold water disease of ayu (Japanese Ministry of Agriculture, Forestry and Fisheries). Each isolate received a unique identifier containing the date of sampling encoded in the “yymmdd” format.

### Multi locus sequence typing

The seven target loci as well as the PCR and sequencing protocols are listed in Additional file 2. The sequences were assembled using Phred/Phrap/Consed [30], verified manually to ensure high quality, and deposited in Genbank (GenBank:KC203886-KC204683). In keeping with MLST standards [31,32], arbitrary numbers served for unambiguous identification of the AT (particular alleles at particular loci) and ST (unique combinations of AT at the seven loci). To facilitate large scale studies the following changes were made compared to the initial study [17]: PCR amplification was performed with an optimized touchdown protocol; 5’ extensions were added to the PCR primers to allow sequencing of the 7 loci with the same pair of primers; and AT attribution was based on slightly shortened sequences. The data, including the genotypes from [17,26], are made available in a new version of the *F. psychrophilum* MLST database [33] which uses the BIGSdb system [34].

A tree depicting AT-sharing between ST was built by hierarchical clustering with R; the function “hclust” using the single link aggregation method and drawn with the R package “ape”. The eBURSTv3 software with default settings [35] was used to obtain a “population snapshot” highlighting the relationships between closely related ST. Clonal complexes (CC) were defined using the criterion of single locus variant (SLV) connections between ST. Analyses of nucleotide diversity, including analysis of molecular variance [36], were coded in R. Nucleotide diversity was computed on pairwise comparisons between unique ST rather than between isolates to minimize the impact of sampling biases; as a consequence, the values were slightly higher than those initially reported in Nicolas et al. [17].

## Results and discussion

### Sampling of *F. psychrophilum* diversity at whole-country and model river levels

We analyzed bacterial isolates representative of two different geographical levels. In addition to 6 MLST profiles already available [17], we genotyped a first set of 34 *F. psychrophilum* isolates collected between 1993 and 2005 from a variety of locations all over Japan to account for the diversity at the country level. Our second set consisted of 80 isolates obtained for this study between August 2005 and December 2006 from a single model river in which fish were captured irrespective of their disease status to minimize sampling biases. This strategy allowed retrieving isolates from 6 fish species, as well as 4 isolates from water samples and 1 from the case of a caddisfly (order *Trichoptera*). This raises the number of MLST profiles available to study the *F. psychrophilum* population structure in Japan to 120 and the number of sampled host fish species to 15. Table 1 provides a detailed summary of the data. These could be compared to 110 other MLST profiles from the rest of the world [17,26].

**Table 1:** **MLST profiles and background information for the 120 *F. psychrophilum *isolates from Japan**

**Isolate**^**a**^	**ATs**^**b**^	**ST**^**c**^	**Prefecture**^**d**^	**Host**^**e**^	**Tissue**	**Year**
FPC 837*	5, 4, 4, 4, 2, 4, 4	ST5	Tokushima	*P. altivelis* (ayu)	Kidney	1987
FPC 840*	5, 4, 4, 4, 2, 4, 4	ST5	Tokushima		Kidney	1987
PH9351	19,24,19, 5, 8, 1, 8	ST45	Hiroshima		Ovary	1993
CS-1	19,24,19, 5, 8, 1, 8	ST45	Gifu		Kidney	1995
96-4	5,24, 4, 4, 2, 4, 4	ST52	Gifu		Kidney	1996
PH-0003	19,24,19, 5, 8, 1, 8	ST45	Hiroshima		Kidney	2000
SG011227	5,24, 2, 6, 8, 1, 8	ST56	Shiga		Kidney	2001
PH-0209	19,24,19, 6, 8, 1, 8	ST48	Hiroshima		Kidney	2002
AK-0527	19,19,19, 2,13,29,28	ST53	Kyoto		Lower jaw	2005
AK-0536	19,19,19, 2,13,29,28	ST53	Kyoto		Lower jaw	2005
AK-0531	19,19,19, 2,13,29,28	ST53	Kyoto		Kidney	2005
AK-05137	19,19,19, 2,13,29,28	ST53	Kyoto		Skin lesion	2005
KU050822-1	5,24, 4, 4, 2, 4, 4	ST52	Shiga (MR)		Skin lesion	2005
KU050822-3 ~ 4	5,24, 4, 4, 2, 4, 4	ST52(2)	Shiga (MR)		Skin lesion	2005
KU051024-5	5,24, 4, 4, 2, 4, 4	ST52	Shiga (MR)		Gill	2005
KU051024-6	5,24, 4, 4, 2, 4, 4	ST52	Shiga (MR)		Coelomic fluid	2005
KU051024-7	5,24, 4, 4, 2, 4, 4	ST52	Shiga (MR)		Coelomic fluid	2005
KU051024-8 ~ 11	5,24, 2, 6,10, 1, 8	ST65(4)	Shiga (MR)		Egg	2005
KU051024-12 ~ 13	5,24, 4, 4, 2, 4, 4	ST52(2)	Shiga (MR)		Egg	2005
KU051024-14 ~ 17	5,24, 4, 4, 2, 4, 4	ST52(4)	Shiga (MR)		Egg	2005
KU060427-1	5,24, 4, 4, 2, 4, 4	ST52	Shiga (MR)		Gill	2006
KU060626-1 ~ 3	19,24,19, 6, 8, 1, 8	ST48(1)	Shiga (MR)		Kidney	2006
	5,24, 4, 4, 2, 4, 4	ST52(2)	-		-	-
KU060626-4 ~ 6	5,24, 2, 6, 8, 1,27	ST49(3)	Shiga (MR)		Kidney	2006
KU060626-7 ~ 9	5,24, 4, 4, 2, 4, 4	ST52(3)	Shiga (MR)		Kidney	2006
KU060626-10 ~ 12	5,24, 4, 4, 2, 4, 4	ST52(3)	Shiga (MR)		Kidney	2006
KU060626-13 ~ 15	5,24, 4, 4, 2, 4, 4	ST52(3)	Shiga (MR)		Kidney	2006
KU060626-16 ~ 18	5,24, 4, 4, 2, 4, 4	ST52(3)	Shiga (MR)		Kidney	2006
KU060626-19 ~ 21	19,24,19, 6, 8, 1, 8	ST48(2)	Shiga (MR)		Kidney	2006
	5,24, 2, 5, 8, 1,35	ST67(1)	-		-	-
KU060626-22 ~ 24	5,24, 4, 4, 2, 4, 4	ST52(3)	Shiga (MR)		Kidney	2006
KU060626-25 ~ 27	5,24, 4, 4, 2, 4, 4	ST52(3)	Shiga (MR)		Kidney	2006
KU060626-28 ~ 36	19,24,19, 6, 8, 1, 8	ST48(1)	Shiga (MR)		Kidney	2006
	5,24, 4, 4, 2, 4, 4	ST52(7)	-		-	-
KU060626-37 ~ 39	19,24,19, 6, 8, 1, 8	ST48(3)	Shiga (MR)		Kidney	2006
KU060626-40 ~ 42	5,24, 4, 4, 2, 4, 4	ST52(3)	Shiga (MR)		Kidney	2006
KU060626-43 ~ 46	19,24,19, 6, 8, 1, 8	ST48(1)	Shiga (MR)		Kidney	2006
	5,24, 4, 4, 2, 4, 4	ST52(3)	-		-	-
KU060626-57	5,24, 4, 4, 2, 4, 4	ST52	Shiga (MR)		Skin lesion	2006
KU060626-59	19,24,19, 6, 8, 1, 8	ST48	Shiga (MR)		Skin lesion	2006
KU060920-5 ~ 6	5,24, 4, 4, 2, 4, 4	ST52(2)	Shiga (MR)		Gill	2006
KU060920-7 ~ 8	5,24, 4, 4, 2, 4, 4	ST52(2)	Shiga (MR)		Gill	2006
KU060920-19	5,24, 4, 4, 2, 4, 4	ST52	Shiga (MR)		Milt	2006
OH-0224	2,30,20, 2,22,22,23	ST54	Hiroshima	*O. masou* subsp. (resp. *yamame*, *amago x*2, and *biwamasu*)	Gill	2003
96-1	17,26,18, 9,19,24,24	ST43	Gifu		Kidney	1996
OH-0519	2,30,20, 2,23,22,23	ST55	Hiroshima		Kidney	2005
SG030207	15,24,10, 3, 2,12,11	ST40	Shiga		Kidney	2003
FPC 813*	2, 8, 2, 2, 2,10, 2	ST17	Tokyo	*O. mykiss* (rainbow trout)	Kidney	1992
FPC 814*	2, 8, 2, 2, 2,10, 2	ST17	Tokyo		Kidney	1992
CS-3	14,23, 4,10, 6,22, 2	ST39	Gifu		Kidney	1995
SG950607	2, 8, 2, 2, 2, 2, 2	ST10	Shiga		Kidney	1995
SG010808	2,29,15,12,17,27,26	ST47	Shiga		Kidney	2001
OH-0203	2, 8, 2, 2, 2, 2, 2	ST10	Hiroshima		Kidney	2002
0312	7,28, 4, 5,21,28,21	ST50	Yamanashi		Kidney	2003
y-2	2, 8, 2, 2, 2, 2, 2	ST10	Iwate		Kidney	2005
y-3	4,25, 4, 5,18,23,23	ST42	Iwate		Kidney	2005
FPC 830*	4, 7, 6, 5, 6, 8, 4	ST13	Miyagi	*O. kisutch* (coho salmon)	Kidney	1990
FPC 831*	11,18, 7, 5,14,18,17	ST30	Iwate		Peduncle	1990
FPM960724	11,18, 7, 5,14,18,17	ST30	Miyagi		not available	1996
FPM960726	4, 7, 6, 5, 6, 5, 4	ST9	Miyagi		Kidney	1996
x-2	11,18, 7, 5,14,18,17	ST30	Iwate		Kidney	2005
SG020617	16, 4,17, 5,10,12,22	ST41	Shiga	*S. leuc*. (*iwana*)	Kidney	2002
SG980216	18,27,10, 7,20,25,25	ST44	Shiga	*H. nipponensis* (*wakasagi*)	Egg	1998
SG010619	5,24, 4, 4, 2, 4, 4	ST52	Shiga		Kidney	2001
SG040302	20,31,19, 2,23,30,28	ST57	Shiga		Kidney	2004
GM2127	4,28, 8,11, 1,26, 4	ST46	Gunma	*T. hakonensis* (*ugui*)	Skin lesion	1999
CH-9401	5,24, 8, 6, 8, 1, 8	ST51	Hiroshima	*C. carpio* (*koi*)	Skin lesion	1994
KU061226-2 ~ 3	24,21,15, 1, 8,34,33	ST63(2)	Shiga (MR)	Cyprinid fish	Gill	2006
CH-0411	5,24, 8, 6, 8, 1, 8	ST51	Hiroshima	*C. carassius* (*funa*)	Kidney	2004
KU061226-1	25,35,10, 2,16, 1, 4	ST64	Shiga (MR)	*P. jouyi* (*takahaya*)	Gill	2006
PH-9348	21,32,10, 7,24,31,29	ST58	Hiroshima	*Z. platypus* (*oikawa*)	Kidney	1993
ZH-0001	5,24, 8, 6, 8, 1, 8	ST51	Hiroshima		Kidney	2000
KU051024-4	5,24, 4, 4, 2, 4, 4	ST52	Shiga (MR)	*T. bre.* (*numachichibu*)	Gill	2005
KU051128-1	1,15,16, 5,19,35,34	ST66	Shiga (MR)	*Gymnogobius* sp.	Gill	2005
KU051128-4	23,29,11,13,13, 5,1	ST60	Shiga (MR)	*L. rei.* (*sunayatsume*)	Gill	2005
KU051128-8	23,29,11,13,13, 5,1	ST60	Shiga (MR)	River water	not applicable	2005
KU051128-10	22,31, 4,10,19,32,3	ST59	Shiga (MR)		not applicable	2005
KU060920-2	5,24, 4, 4, 2, 4, 4	ST52	Shiga (MR)		not applicable	2006
KU060920-4	5,24, 4, 4, 2, 4, 4	ST52	Shiga		not applicable	2006
KU061128-1	5, 34, 8, 1, 26, 26, 32	ST62	Shiga (MR)		not applicable	2006
KU051128-6	8, 33, 8, 1, 25, 33, 31	ST61	Shiga (MR)	Case of caddisfly	not applicable	2005

^a^ Isolate identifier. Isolates are ordered by host fish species and then by sampling year; * indicates isolates already included in Nicolas et al. [17]. When several isolates have been collected from the same individual fish their identifiers are reported in a condensed format (i.e. KU060626-40 ~ 42 stands for KU060626-40, KU060626-41 and KU060626-42).

^b^ Allele types (AT) for the seven loci ordered *trpB*, *gyrB*, *dnaK*, *fumC*, *murG*, *tuf* and *atpA*.

^c^ Sequence type (ST). When several isolates were collected from the same individual fish the number in parentheses indicates the frequency of each ST. If different ST were found in the same fish, each ST is reported on a separate line but information on prefecture, tissue and year are not repeated (replaced by -).

^d^ Geographical origin of the isolate in Japan. (MR) indicates an isolate from the model river in the Shiga prefecture.

^e^ The host fish species or environmental sample source. The common name used in Japan is given in parentheses. Genus and sometimes species names have been abbreviated in this column. Full species names: *Plecoglossus altivelis*, *Oncorhynchus masou*, *Oncorhynchus mykiss*, *Oncorhynchus kisutch*, *Salvelinus leucomaenis*, *Hypomesus nipponensis*, *Trybolodon hakonensis*, *Cyprinus carpio*, *Carassius carassius*, *Phoxinus jouyi*, *Zacco platypus*, *Tridentiger brevispinis*, and *Lethenteron reissneri*.

### Population structure at the country level

**Figure 1:**
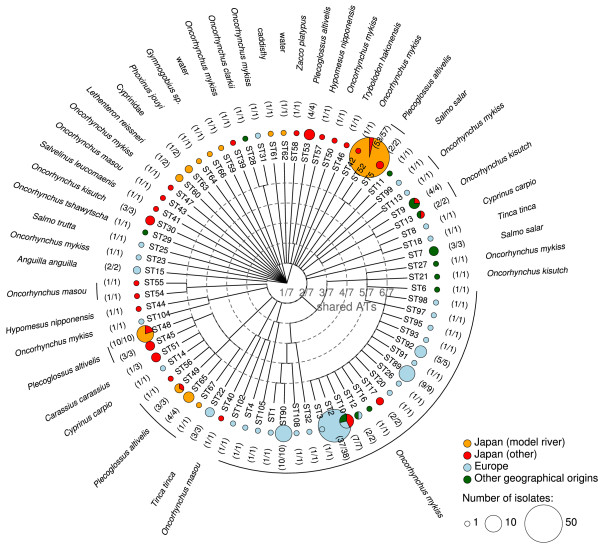
**Genetic relationships, geographical origins and host fish species of the 230 *F. psychrophilum *isolates with MLST profiles available.** The tree was built based on the number of shared allele types (AT) using hierarchical clustering and a single link aggregation criterion. This choice ensures a relative robustness with respect to recombination as well as consistency with the eBURST diagram and the definition of clonal complexes. The circle associated with each sequence type (ST) reflects its number of occurrences (size) and geographical origin (color). The most frequent fish host for each ST is reported on the side together with its relative frequency (between parentheses).

Our MLST data reveal a high genetic diversity of *F. psychrophilum* in Japan. The total number of different ST in the Japanese isolate collection amounts to 35, among which 32 are only reported in Japan (Figure 1). At the nucleotide level, these 35 ST harbor a pairwise nucleotide diversity of 5.4 Kbp^-1^ comparable to the 5.6 Kbp^-1^ found in the 230 isolates representative of worldwide diversity. This indicates that the structure of the *F. psychrophilum* population is at most only marginally linked to geographical origin. Indeed, in our data, as much as 88% of the nucleotide sites that are polymorphic in Europe are also polymorphic in Japan.

**Figure 2:**
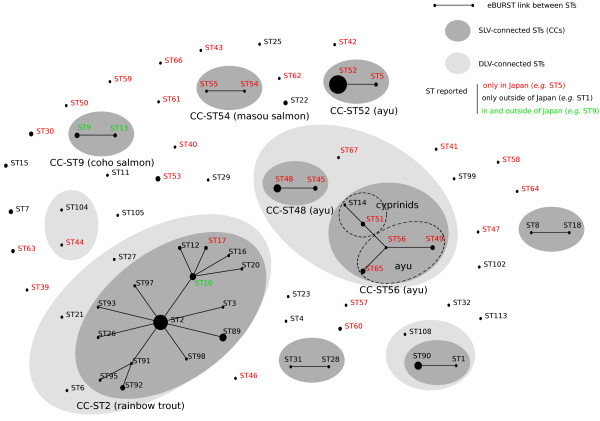
**The eBURST population snapshot of the *F. psychrophilum *MLST data.** The relationships between the 230 isolates with the MLST profile available are depicted in this diagram.

Many of the ST that were sampled several times are associated with the defined host fish (Figures 1 and 2). The ST of most Japanese isolates (76%) could indeed be classified into six CC and the ST from a same CC also tended to infect similar host fish. Each CC is named after a representative ST. These six CC are the following: CC-ST52 (59 isolates) from ayu; CC-ST48 (13 isolates) from ayu; CC-ST56 (12 isolates) from ayu and cyprinids; CC-ST2 (3 isolates) from rainbow trout; CC-ST9 (2 isolates) from coho salmon; and CC-ST54 (2 isolates) from masou salmon. Of note, CC-ST48 and CC-ST56 that both infect ayu are connected by a double locus variant (DLV) link and therefore probably share a recent common ancestor, even though here they are distinguished using the stringent operational definition of CC based on the SLV links.

Analysis of the molecular variance confirmed that the association between genotype and the host fish in Japan was highly statistically significant and showed that it explains as much as 44.2% of the genetic variance. This level was slightly lower but close to the 51.3% reported at the worldwide level by Nicolas et al. [17] and to the 48.3% computed here on the new set of 230 isolates.

At least two separate lineages (CC-ST52 and CC-ST48/CC-ST56) infect this fish species, a fact that is important for the future treatment and prevention of BCWD in ayu. Strains in these three CC showed various levels of virulence against ayu in bath infection challenges and strains from CC-ST48/CC-ST56 generally seem more virulent than those from CC-ST52 [14]. Of note, CC-ST56 also infects cyprinids in Japan and [14] reported that ST-51 isolates from cyprinids cause mortality in ayu by bath infection challenge, providing experimental support for the possibility of transfer between carp and ayu in CC-ST56.

### Footprints and possible mechanisms of transcontinental dissemination

The absence of clear genetic signal reflecting the geography indicates mixing between *F. psychrophilum* populations located on different continents. Such a deep effect on the whole population probably involved recurrent natural dissemination before the development of commercial exchanges. This may seem surprising for a fish-pathogenic bacterium found in freshwater that one can believe limited by watersheds. However, long-distance dissemination events do not need to be frequent to have a substantial impact on genetic structure: a few migrants per generation in the entire population have dramatic homogenizing consequences in idealized models [37,38]. Potential mechanisms include fish and bird migrations but airborne dissemination may be another possibility. Indeed, *F. psychrophilum* is able to survive in pure water [39] and freshwater bacteria -including *Flavobacteriaceae*- were recently sampled in the upper troposphere [40]. Alternatively, genetic mixing can involve a succession of more frequent small-distance steps. The DLV link between ST44 sampled in Japan from *H. nipponensis* and ST104 sampled in France from rainbow trout might be a footprint of natural dissemination. Both ST have been sampled only once and are not connected to other genotypes, which suggests that the ST44/ST104 lineage infects farmed fish only sporadically and should thus not be prone to dissemination by human activities.

In sharp contrast, the three ST also reported in other countries (ST9, ST10 and ST13) are associated to rainbow trout and coho salmon. These are two fish species with wide geographical distribution and whose eggs and broodfish have been imported to Japan (vertical transmission from parent to offspring via eggs is highly suspected [41]). Two other ST that we found in Japan are connected by SLV links to ST found abroad: ST17, sampled twice from rainbow trout, is linked to the main rainbow trout clonal complex (CC-ST2); and CC-ST56, infecting ayu and cyprinids in Japan, is linked to ST14 sampled from a carp in Germany. In both cases, commercial exchanges may be invoked to explain these SLV links between Japanese and European isolates. Namely, carps have been traded between Japan and Europe, and rainbow trout have been imported from North America to both Japan and Europe. Of note, the diversity of CC-ST48/CC-ST56 found in Japan is a strong indication that dissemination of this lineage occurred from Japan to Europe rather than the contrary; altogether we found no indication suggesting that strains infecting ayu might have been recently imported from foreign sources.

### Population structure in the model river

Extensive sampling of a model river unveils an unanticipated level of diversity in a restricted geographical area. We found 12 different ST in the model river and a general pattern of polymorphism surprisingly similar to that of the whole country and worldwide levels. Namely, the average pairwise nucleotide difference between the ST was 4.8 Kbp^-1^ and fish host accounted for 51.2% of the molecular variance.

The isolates from the model river include representatives of the three clonal complexes reported in ayu. The majority of ayu isolates belonged to ST52 and this ST was observed over several seasons (i.e., August 2005, October 2005, April 2006, June 2006, and September 2006). Simultaneously, strains displaying ST52 were also isolated from numachichibu and a water sample suggesting contamination by ayu. A geographical population structure could explain the dominance of CC-ST52 in the model river not seen at the country level. However, at least two other factors may contribute to this difference: a temporal evolution of relative frequencies, since most isolates sampled at the country level were collected years before the sampling of the model river; and the fish sampling strategy, since sampling in the model river was performed irrespectively of disease symptoms and CC-ST52 seems less virulent [14]. Seven ST (ST59, ST60, ST61, ST62, ST63, ST64, and ST66) not connected to those found in ayu were retrieved from river water, a case of caddisfly, and several local fish. These strains were isolated in November 2005, November 2006 and December 2006, when ayu are absent from the river due to their one-year life cycle ending after spawning in the autumn. Six of these strains (no data available on ST66) showed no pathogenicity against ayu [14]. These ST may have gone unnoticed if other fish species and the environment had not been sampled.

Importantly, co-infection of the same individual fish by different lineages was detected four times in ayu (Table 1). This observation could have a practical interest in the context of disease prevention or treatment but could also have important evolutionary implications. In particular, co-infection might promote evolution towards higher virulence [36], in particular if distinct transmission modes –horizontal and vertical- are involved [42]. The local diversity of *F. psychrophilum* genotypes detected in the model river, which goes down to the level of the individual host fish, also certainly contributes to explain the very high recombination rate detected in the species on the basis of MLST data [17,43]. Indeed, a greater local diversity provides more opportunities to exchange alleles and therefore directly increases the apparent recombination rate and the actual genetic mixing.

## Abbreviations

BCWD: Bacterial cold water disease; PCR: Polymerase chain reaction; PBS: Phosphate buffered saline; MLST: Multilocus sequence typing; CC: Clonal complex; ST: Sequence type; AT: Allele type; SLV: Single locus variant; DLV: Double locus variant.

## Competing interests

The authors declare that they have no competing interests.

## Authors’ contributions

EFN performed fish sampling in the model river, bacterial isolation, and contributed to sample processing, data analysis and writing of the manuscript. CCD contributed to the development and optimization of the MLST scheme and to sample processing. JFB and ME contributed to data analysis and presentation of the results. ED initiated the project and contributed to data analysis. PN analyzed the data and wrote the manuscript. All authors read and approved the final manuscript.

## Supplementary Material

Additional file 1 **Isolates representative of diversity at the country level.** Institutions that provided these isolates and previous publications on these isolates are listed [11,14,16,44-47]. Click here for file

Additional file 2 **MLST scheme.** PCR and sequencing primers for the seven loci as well as the corresponding experimental protocols are provided. Click here for file
