# Supplementary material for: Population structure of the fish pathogen Flavobacterium psychrophilum at whole-country and model river levels in Japan
Source: Vet Res. 2013 May 17;44(1):34. doi: 10.1186/1297-9716-44-34 (PMC3660162; doi:10.1186/1297-9716-44-34)
Supplement: Additional file 1 — Isolates representative of diversity at the country level. Institutions that provided these isolates and previous publications on these isolates are listed [11,14,16,44-47]. [file 1297-9716-44-34-S1.doc]

Additional File 1 Isolates representative of diversity at country level.

| Isolate | | Institution | | Reference |
| --- | --- | --- | --- | --- |
| y-2 | | Iwate Prefectural Inland Fisheries Technology Center | | [14] |
| y-3 | |  |  |  |
| x-2 | |  |  |  |
| FPM960724 | | Miyagi Prefecture Fisheries Technology Institute | | [14,44] |
| FPM960726 | |  |  |  |
| GM2127 | | Gunma Prefectural Fisheries Experimental Station | | [14,44] |
| 0312 | | Yamanashi Prefectural Fisheries Technology Center | | [14] |
| CS-1 | | Gifu Prefectural Research Institute for Freshwater Fish and Aquatic Environments | | [14,44] |
| 96-4 | |  |  | [14] |
| 96-1 | |  |  | [14] |
| CS-3 | |  |  | [14] |
| SG011227 | | Shiga Prefectural Fisheries Experimental Station | | [11,14,45] |
| SG030207 | |  |  | [14,44,46] |
| SG950607 | |  |  | [14,44] |
| SG010808 | |  |  | [14,44] |
| SG020617 | |  |  | [14,44] |
| SG980216 | |  |  | [14,44] |
| SG010619 | |  |  | [14,44] |
| SG040302 | |  |  | [14] |
| KU060920-4 | | Kinki University, Dept of Fisheries | |  |
| AK-0527 | | Fisheries Technology Department of Kyoto Prefectural Agriculture, Forestry, and Fisheries Technology Center | | [14] |
| AK-0536 | |  |  |  |
| AK-0531 | |  |  |  |
| AK-05137 | |  |  |  |
| PH9351 | | Hiroshima Prefectural Technology Research Institute | | [14,16,44] |
| PH-0003 | |  |  | [47] |
| PH-0209 | |  |  | [14] |
| OH-0224 | |  |  | [14,47] |
| OH-0519 | |  |  | [14,47] |
| OH-0203 | |  |  | [14,47] |
| CH-9401 | |  |  | [44] |
| CH-0411 | |  |  | [14,47] |
| PH-9348 | |  |  | [44] |
| ZH-0001 | |  |  | [14,47] |
|  |  | |  | |
