# Supplementary material for: Population structure of the fish pathogen Flavobacterium psychrophilum at whole-country and model river levels in Japan
Source: Vet Res. 2013 May 17;44(1):34. doi: 10.1186/1297-9716-44-34 (PMC3660162; doi:10.1186/1297-9716-44-34)
Supplement: Additional file 2 — MLST scheme. PCR and sequencing primers for the seven loci as well as the corresponding experimental protocols are provided. [file 1297-9716-44-34-S2.doc]

**Additional File 2** MLST scheme.

| Locus | Forward primer (5'-3') | Reverse primer (5'-3') | Length^a^ |
| --- | --- | --- | --- |
| *trpB* | CAGGAAACAGCTATGACCAAGATTATGTAGGCCGCCC | TGTAAAACGACGGCCAGTTGATAGATTGATGACTACAATATC | 789 bp |
| *gyrB* | CAGGAAACAGCTATGACCGTTGTAATGACTAAAATTGGTG | TGTAAAACGACGGCCAGTCAATATCGGCATCACACAT | 1077 bp |
| *dnaK* | CAGGAAACAGCTATGACCAAGGTGGAGAAATTAAAGTAGG | TGTAAAACGACGGCCAGTCCACCCATAGTTTCGATACC | 873 bp |
| *fumC* | CAGGAAACAGCTATGACCCCAGCAAACAAATACTGGGG | TGTAAAACGACGGCCAGTGGTTTACTTTTCCTGGCATGAT | 750 bp |
| *murG* | CAGGAAACAGCTATGACCTGGCGGTACAGGAGGACATAT | TGTAAAACGACGGCCAGTGCATTCTTGGTTTGATGGTCTTC | 681 bp |
| *tuf* | CAGGAAACAGCTATGACCGAAGAAAAAGAAAGAGGTATTAC | TGTAAAACGACGGCCAGTCACCTTCACGGATAGCGAA | 795 bp |
| *atpA* | CAGGAAACAGCTATGACCCTTGAAGAAGATAATGTGGG | TGTAAAACGACGGCCAGTTGTTCCAGCTACTTTTTTCAT | 834 bp |

^a^ Length of the target sequence.

forward sequencing primer: 5’-CAGGAAACAGCTATGACC-3’

reverse sequencing primer: 5’-TGTAAAACGACGGCCAGT-3’
